# Paravertebral crystal deposition disease: a retrospective study of clinical presentation, prevalence, and CT imaging findings

**DOI:** 10.1007/s00256-025-04874-w

**Published:** 2025-01-16

**Authors:** Taro Takeda, Mieko Takasugi, Kotaro Yoshida

**Affiliations:** 1 Department of Radiology, Matsunami General Hospital, 185-1 Dendai, Kasamatsu-Cho, Gifu, Hashima-Gun 501-6062 Japan; 2 Department of Radiology, Fukui Prefectural Hospital, 2-8-1 Yotsui, Fukui City, 910-8526 Japan

**Keywords:** Paravertebral crystal deposition, Calcification, Spine, Inflammation, Computed tomography

## Abstract

**Objectives:**

Paravertebral crystal deposition disease, characterized by the deposition of crystals around the vertebral bodies leading to acute inflammation and pain, is a condition that remains largely unrecognized. This study aims to elucidate the prevalence, clinical features, and CT findings associated with this disease.

**Methods:**

We retrospectively analyzed 14,839 consecutive patients who underwent chest and/or abdominal CT (September 2017 to September 2024) owing to chest, abdominal, or back pain. Cases demonstrating paravertebral calcification with a surrounding soft tissue density of ≥ 5 mm were identified and further evaluated.

**Results:**

Twenty-four cases of paravertebral crystal deposition disease were identified, with a prevalence of 0.16% (95% CI: 0.10, 0.24). The mean age was 46.2 years, with a female predominance (*n* = 15, 63%). Back pain was the most common presenting symptom (*n* = 15, 63%). Calcifications were primarily located at the lower thoracic and upper lumbar spine (Th6/7-L1/2) in 18 cases (75%) and in the anterior median to anterior right region of the vertebral body in 21 cases (86%). Mean of maximum CT values of the crystal deposition was approximately 800 HU; in follow-up cases, the depositions either resolved or exhibited morphological changes.

**Conclusions:**

This study adds to the current knowledge base by identifying a 0.16% prevalence of paravertebral crystal deposition disease in patients with torso pain—often overlooked in clinical practice, primarily affecting middle-aged women. CT imaging shows calcifications mainly in the lower thoracic and upper lumbar spine. Considering this self-limiting disease in differential diagnoses can improve diagnostic accuracy and patient management.

**Supplementary Information:**

The online version contains supplementary material available at 10.1007/s00256-025-04874-w.

## Introduction

Crystal deposition disease notably affects various joints throughout the body, including first metatarsophalangeal joint, knee, and shoulder, leading to symptoms associated with localized acute inflammation [1]. Monosodium urate, calcium pyrophosphate crystals, and basic calcium phosphate crystals are recognized as the primary constituents of this disease, with the type of crystal varying depending on the location of the deposition [2–5]. Crystal deposition can also occur in the spinal region, such as pediatric intervertebral disc calcification, yellow ligament calcification, and longus colli muscle calcification [6–8].

Limited case reports have documented instances of acute inflammation due to crystal deposition around the vertebral bodies, separate from the conditions mentioned above [9–11]. This condition presents with trunk pain as the primary symptom and is typically detected and diagnosed through CT imaging [9]. With the widespread use of CT scans, the diagnosis of paravertebral crystal deposition disease is expected to increase. However, there are no comprehensive reports on the incidence, clinical presentation, locations, or imaging characteristics of crystal deposition around the vertebra. This study aims to retrospectively determine the incidence and imaging characteristics of paravertebral crystal deposition disease among patients presenting with trunk pain who underwent CT scans at a single institution.

## Materials and methods

### Case selection and inclusion criteria

This retrospective study was approved by the institutional review board of Matsunami General Hospital (Matsu-Irin 597). The need for written informed consent was waived owing to the retrospective nature of the study. This study was conducted at a facility providing primary and secondary care in Japan, which performs approximately 30,000 CT examinations annually. A systematic keyword search of the radiology report database was performed to identify candidate cases within the 194,065 CT examinations conducted between September 2017 and September 2024, where the purpose of the examination included the following: chest, back, lower back, abdominal, epigastric, flank, lateral, and lower abdominal pain. Consequently, a total of 14,839 inpatients and outpatients who underwent chest and/or abdominal CT were selected for this study cohort.

The patient selection was conducted by two radiologists (MT and TT), with 18 and 23 years of experience in body diagnosis, respectively. In cases of disagreement, a consensus was reached to make the final decision. In the absence of established diagnostic criteria for paravertebral crystal deposition disease, we first conducted a thorough review of the existing literature on crystal deposition diseases, including specific studies addressing paravertebral crystal deposition disease [1, 9–15]. Based on the literature review, we defined paravertebral crystal deposition disease on CT as the presence of calcification with a peri-lesional soft tissue density of ≥ 5 mm surrounding the vertebral body. The detection of paravertebral soft tissue density was evaluated using a soft tissue window setting, while the detection of calcifications was assessed by combining soft tissue and bone window settings. Exclusion criteria comprised patients with diseases that could cause inflammation around the vertebral bodies, such as vertebral fractures, pyogenic spondylodiscitis, spondylosis, and neoplastic lesions. Cases potentially meeting this criterion were carefully evaluated and excluded from the study. Additionally, past and follow-up CT images, when available, were reviewed to determine the association between calcification and current inflammatory symptoms. The cervical spine was excluded from the study because calcific tendinitis of the longus colli, which presents with neck pain, is a differential diagnosis for this disease.

### Diagnostic process for the included cases

**Fig. 1:**
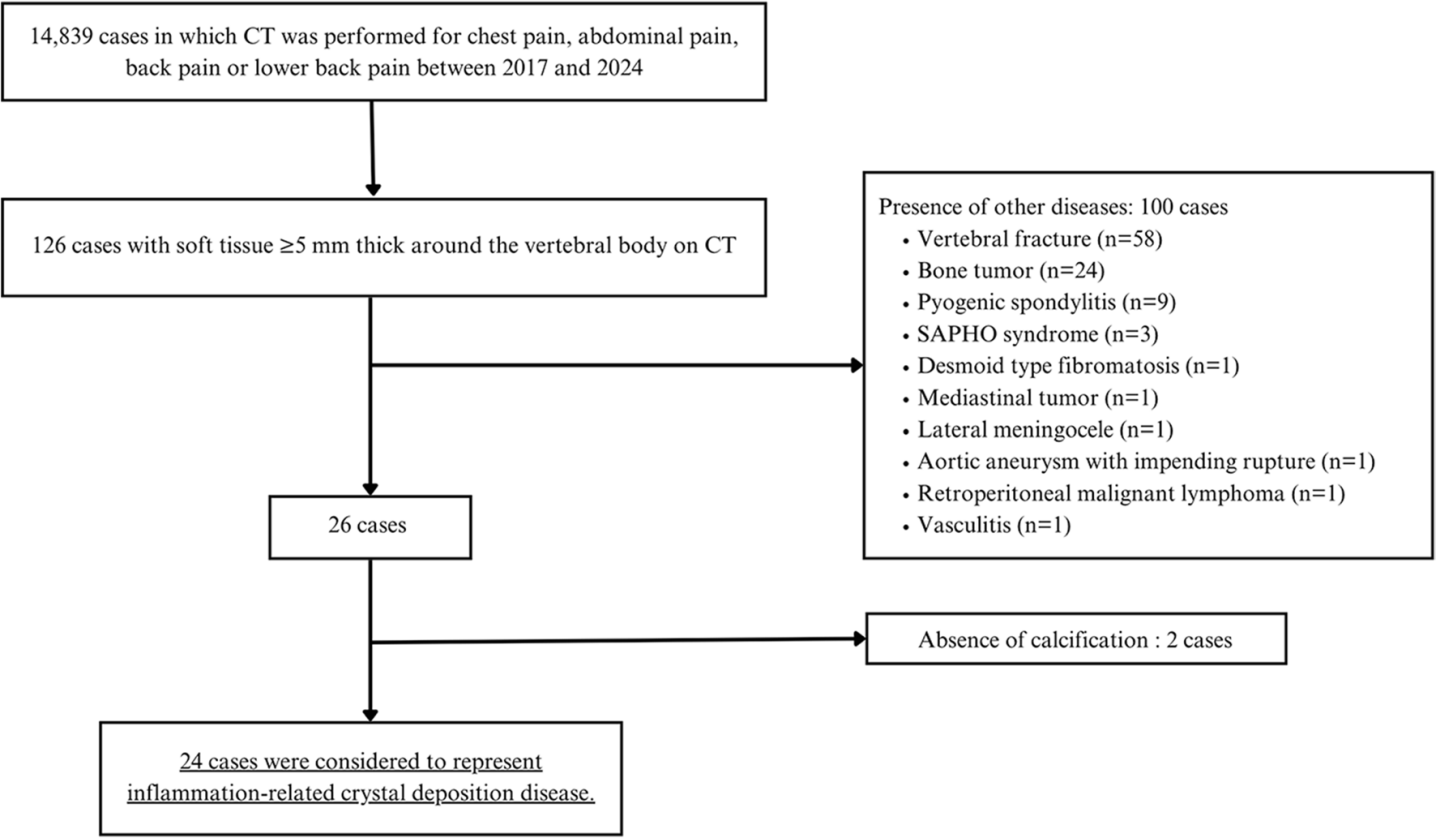
Flowchart showing patient selection and enrollment

The flowchart of patient enrollment is shown in Fig. 1. Among the 14,839 CT scans, 126 cases with soft tissue thickness of ≥ 5 mm around the vertebral bodies were identified. Of the 126 cases with soft tissue density, 100 cases were excluded due to the presence of other aforementioned diseases, and the remaining 26 cases were further assessed for calcification. Two of these 26 cases were further excluded due to the absence of calcification. Finally, 24 cases with calcified lesions coexisting with soft tissue density were ultimately diagnosed as paravertebral crystal deposition disease.

### Image acquisition

Chest and abdominal CT scans were performed using 16- to 256-row multidetector CT scanners (Lightspeed RT XTRA, Optima 660 or Revolution; GE Healthcare). The scanning parameters were as follows: tube voltage of 120 kVp and automatic tube current modulation ranging from 70 to 600 mA with Noise Index of 9.8–13.0, adjusted according to the patient’s body habitus. The rotation time was set between 0.5 and 0.7 s, detector collimation at 0.625 mm, and pitch ranging from 0.984 to 1.375. A uniform slice thickness of 5 mm was utilized for axial images and 2 mm for sagittal images or coronal images, depending on the location of the calcification.

### Clinical information

For cases diagnosed with paravertebral crystal deposition disease, the following data were collected: age, sex, symptoms, interval between symptom onset and CT, referring department, presence of fever or neurological symptoms (numbness or paralysis), and history of spinal diseases. The white blood cell count (WBC) and C-reactive protein (CRP) levels were extracted from the laboratory database. Information on treatment methods and symptom progression after diagnosis was extracted from the electronic medical record system. Previous occurrences of similar symptoms were also documented.

### Image evaluation

The imaging evaluation was conducted by two radiologists (MT and TT), with 18 and 23 years of experience in body CT diagnosis, respectively. Over 70% of cases were prospectively evaluated during routine clinical interpretations, while the remaining cases underwent retrospective review over a 3-month period. CT images were scrutinized for the affected vertebrae, alongside the location and dimensions of paravertebral calcifications. To achieve a comprehensive three-dimensional assessment of the lesions, imaging evaluations were conducted for both sagittal/coronal (depending on the location of the calcification) and axial reconstructed images. The longitudinal position of the calcification was classified into three categories: at the intervertebral disc level, at the vertebral body level, or extending from the vertebral body to the intervertebral disc. The transverse position of the calcification was classified into three categories: the anterior aspect, left side, and right side of the vertebral body (see Supplemental Fig. 1 for definitions used for classification). One radiologist measured the size of the calcification across three axes and determined the maximum CT number of the calcification using a region of interest setting with CT images (window width: 2000 Hounsfield units [HU], window level: 500 HU). The CT number of the calcification was compared to that of the cortical bone of the vertebral spinous process. For cases with follow-up CT images, the progression of calcification was evaluated.

## Results

### Clinical presentation

Among the 14,839 patients who underwent chest and abdominal CT scans due to complaints of torso pain, 24 cases of paravertebral crystal deposition disease were identified, yielding a prevalence of 0.16% (95% CI: 0.10, 0.24). The clinical characteristics of these enrolled patients are presented in Table 1. The mean age of the patients was 46.2 ± 9.1 years (age range: 31–67 years), with a female predominance (women, *n* = 15: men, *n* = 9). The most common presenting symptom was back pain (*n* = 15, 63%), followed by lower back pain (*n* = 4, 17%) and epigastric pain (*n* = 3, 13%). The duration from symptom onset to medical consultation, which typically coincided with the same day CT scan, was a maximum of nine days; notably 75% of patients (*n* = 18) visited the hospital within three days of symptom onset. Fever was recorded in six out of 15 patients with temperature documentation, measured at ≥ 37.5 °C. The majority of patients initially presented to the general medicine or emergency departments, with only one patient consulting orthopedics at the outset.

**Table 1:** Demographic characteristics of 24 patients with paravertebral crystal deposition disease

Case No	Age (in years)	Sex	Symptom	Days from symptom to CT	Body temperature (°C)	WBC (/μL)	CRP (mg/dL)	Initial department
1	62	M	Epigastric pain, back pain	1	36.9	11,000	2.0	Cardiology
2	37	F	Lower back pain	1	37.3	8700	0.3	Cardiology
3	43	M	Right back pain	5	NA	NA	NA	Urology
4	42	F	Left flank pain	2	37.9	11,900	1.2	Internal Medicine
5	44	M	Chest pain, back pain	2	NA	8800	0.7	Cardiology
6	58	F	Right lower back to flank pain	3	36.9	6000	0.4	Internal Medicine
7	39	M	Back pain	4	NA	NA	0.0	Internal Medicine
8	46	F	Back pain	3	NA	5100	1.4	Internal Medicine
9	35	M	Back pain, epigastric pain	2	38.0	10,400	3.2	Internal Medicine
10	53	M	Chest pain	6	36.9	6100	0.3	Cardiology
11	34	F	Back pain	0	37.6	10,100	7.8	Internal Medicine
12	55	F	Upper abdominal pain, lower back pain	0	36.6	12,500	0.4	Emergency Medicine
13	52	F	Back pain	3	36.5	5100	1.4	Emergency Medicine
14	42	F	Right upper quadrant pain	9	NA	7300	1.0	Internal Medicine
15	38	F	Right back pain	2	NA	9900	NA	Internal Medicine
16	50	F	Left back pain	1	37.1	7900	8.6	Internal Medicine
17	42	F	Lower back pain, right abdominal pain	2	35.9	8100	1.5	Internal Medicine
18	31	M	Chest pain, back pain	1	38.0	12,500	0.5	Internal Medicine
19	50	M	Epigastric pain, back pain	1	37.8	8400	0.8	Internal Medicine
20	44	F	Back pain	5	NA	7800	1.9	Orthopedics
21	67	M	Anterior chest pain	2	NA	8600	0.8	Cardiology
22	56	F	Right flank pain	5	NA	6100	3.3	Gastroenterology
23	38	F	Back pain	3	38.0	7900	2.5	Internal Medicine
24	51	F	Back pain	1	37.3	6200	5.3	Urology

*NA*, not assessed; *WBC*, white blood cell count; *CRP*, C-reactive protein; *M*, male; *F*, female

None of the patients reported recent trauma, nor did they exhibit neurological symptoms such as numbness or muscle weakness. Additionally, no prior history of spine-related diseases was noted. Among 22 cases assessed for WBC, six cases (27%) showed elevated levels above 10,000/µL. CRP levels were measured in 22 cases, with 17 cases (77%) showing elevated levels above 0.4 mg/dL.

### Radiologic findings

**Fig. 2:**
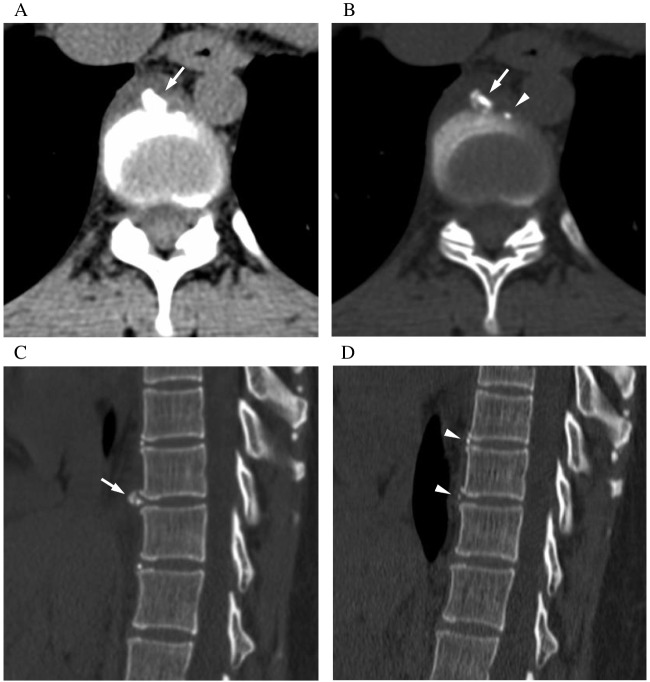
A 37-year-old woman with lower back pain (Patient #2). **A** Axial CT images at the level of Th10/11 intervertebral disc reveals calcification with soft tissue density at the anterior aspect of vertebrae. **B** In axial CT images with bone window setting, the calcification appears heterogeneous with slightly hypodense central portion (arrow). On the left side of this lesion, punctate calcification of the intervertebral disc’s annulus fibrosus is also seen (arrowhead). **C** Sagittal CT image shows paravertebral calcification at the level of Th10/11 adjacent to the calcification of the annulus fibrosus (arrow). **D** In the sagittal CT image obtained 3 months later, the calcification has resolved. Note that there is no interval change in the intervertebral disc calcification at Th9/10, Th10/11, and Th11/12 (arrowheads)

The radiologic characteristics of the enrolled patients with paravertebral crystal deposition disease are summarized in Table 2. The involved vertebrae were most commonly located at the lower thoracic to lumbar levels, with Th6/7 to L1/2 being involved in 18 cases (75%). The longitudinal position of the calcification was primarily at the intervertebral disc level in 16 cases, and it extended across both the intervertebral disc and vertebral body levels in eight cases. No cases were found with calcification confined exclusively to the vertebral body level. The transverse position of the calcifications was distributed as follows: anterior aspect (*n* = 21, 88%) (Fig. 2), left side (*n* = 2, 8%), and right side (*n* = 1, 4%) of the vertebral body.

**Table 2:** Radiologic characteristics of 24 patients with paravertebral crystal deposition disease

Case No	Lesion level	Craniocaudal position of calcification	Axial position of calcification	Size of calcification (mm)^#^	CT value of calcification (HU)
1	Th8/9	IVD	Left	3.4 × 7.6 × 4.9	150
2	Th10/11	IVD	Anterior	6.3 × 6.5 × 6.2	1304
3	Th10/11	IVD and VB	Right	6.1 × 10.0 × 7.4	552
4	Th10/11	IVD	Anterior	4.8 × 3.4 × 5.5	1063
5	Th5/6	IVD	Anterior	2.9 × 2.4 × 6.7	729
6	L1/2	IVD	Anterior	7.0 × 6.3 × 10.5	713
7	Th5/6	IVD	Anterior	6.6 × 3.0 × 4.8	932
8	Th10/11	IVD and VB	Anterior	3.9 × 2.6 × 4.3	542
9	Th8/9	IVD	Anterior	7.1 × 4.9 × 7.9	1404
10	Th7/8	IVD and VB	Anterior	6.3 × 2.4 × 6.8	1383
11	Th4/5	IVD and VB	Anterior	5.2 × 2.5 × 5.5	720
12	Th12/L1	IVD and VB	Anterior	5.2 × 3.2 × 9.5	276
13	Th7/8	IVD	Anterior	3.2 × 2.9 × 5.5	972
14	Th6/7	IVD	Anterior	2.3 × 2.0 × 2.5	583
15	Th6/7	IVD	Anterior	2.3 × 3.1 × 3.6	739
16	Th9/10	IVD	Left	4.8 × 6.1 × 4.7	255
17	L1/2	IVD and VB	Anterior	3.2 × 2.0 × 6.7	280
18	Th1/2	IVD	Anterior	3.1 × 3.7 × 3.7	722
19	Th7/8	IVD and VB	Anterior	4.2 × 5.5 × 10.0	909
20	Th8/9	IVD	Anterior	8.1 × 4.6 × 12.0	1807
21	Th1/2	IVD	Anterior	3.3 × 3.8 × 5.0	909
22	Th9/10	IVD and VB	Anterior	15.5 × 4.3 × 11.2	1005
23	Th9/10	IVD	Anterior	7.3 × 4.7 × 5.1	1062
24	Th9/10	IVD	Anterior	6.2 × 5.8 × 7.5	493

*IVD*, intervertebral disc; *VB*, vertebral body; *CRP*, C-reactive protein: *HU*, hounsfield unit; *Th*, thoracic vertebrae; *L*, lumbar vertebrae

^#^Diameter is presented in the following order: width × length × height

**Fig. 3:**
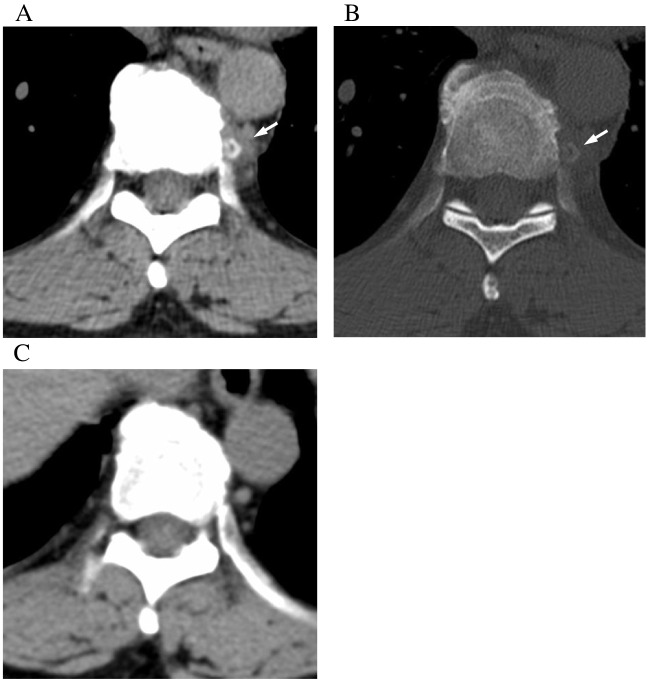
50-year-old women with back pain (Patient #16). **A** CT image setting shows soft tissue density and central paravertebral calcification on the left side of the vertebral body at the level of the Th 9/10 intervertebral disc (arrows). The paravertebral calcification appears heterogeneous. **B** On CT images with bone window setting, the paravertebral calcification demonstrates lower attenuation compared to the cortical bone of vertebrae. The CT value measurement indicated that the maximum value of the calcification was 255 HU. **C** On the CT scan obtained 3 years later, the calcification has resolved

The mean dimensions of the paravertebral calcifications were as follows: transverse diameter, 5.3 ± 2.7 mm; anteroposterior diameter, 4.3 ± 1.9 mm; and craniocaudal diameter, 6.6 ± 2.5 mm. Mean maximum CT values of the crystal deposition were 812.7 ± 399.8 HU compared to 1294.0 ± 195.1 HU for the cortical bone of the spinous processes. In 22 of 24 cases (92%), the CT values of the crystal deposition were lower than those of the cortical bone of the spinous processes (Fig. 3).

### Clinical course

**Fig. 4:**
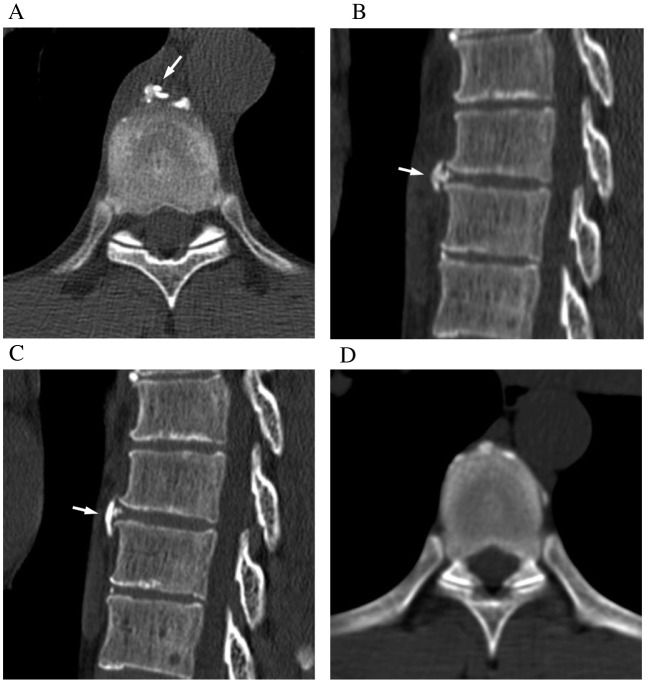
A 35-year-old man with back pain and epigastric pain (Patient #9). **A** Axial CT images with bone window setting shows the paravertebral calcification in the anterior aspect vertebrae at the level of Th 8/9 intervertebral disc. **B** Sagittal CT image reveals an elongated crescent-shaped calcification adjacent to the calcification of the annulus fibrosus (arrow). The paravertebral calcification demonstrates lower attenuation than the calcification of the cortical bone of the vertebral arch. **C** Sagittal CT image obtained 1 month later reveals a craniocaudal expansion of the paravertebral calcification (arrow). **D** CT scan obtained 4 years prior shows no evidence of paravertebral calcification

Detailed information on treatment and subsequent clinical course for all patients is presented in Table 3. The 24 cases included in this study were managed in clinical practice under the suspicion of this condition. Among these 24 cases, 19 were treated with non-steroidal anti-inflammatory drugs, three with acetaminophen, and two were observed without any treatment. None of the cases required further intervention. Follow-up was available for 14 patients, all of whom exhibited symptom improvement. Follow-up CT scans were performed for nine patients; in three cases, the calcifications had completely resolved, while in four, the calcifications had decreased in size (Figs. 2 and 3). In the remaining two cases, the calcifications remained stable, without increase or decrease, although morphological changes were observed on CT (Fig. 4). One patient (Case 3) exhibited a recurrent soft tissue density with calcification at the same level across two separate occasions. In another case (Case 16), a soft tissue density with calcification was observed at the Th3/4 level 8 years ago. Both events occurred prior to the current analysis period.

**Table 3:** Treatment and subsequent course of 24 patients with paravertebral crystal deposition disease

Case No	Treatment	Subsequent symptoms	Change of calcification
1	NSAIDs, antibiotics	Improved by the next day	Resolved
2	NSAIDs	Improved within a few days	Resolved
3	NSAIDs	No follow-up visit	No follow-up image
4	NSAIDs	Improved by the next day	No follow-up image
5	NSAIDs	Improved at 1-week follow-up	No follow-up image
6	NSAIDs	Improved by the next day	Decreased
7	No medication	No follow-up visit	No follow-up image
8	NSAIDs	No follow-up visit	No follow-up image
9	NSAIDs	Improved at 2-week follow-up	Not resolved^#^
10	NSAIDs	Improved within a few days	No follow-up image
11	NSAIDs	Improved within a few days	No follow-up image
12	NSAIDs	No follow-up visit	No follow-up image
13	NSAIDs	Improved at 1-week follow-up	No follow-up image
14	No medication	No follow-up visit	No follow-up image
15	Acetaminophen	No follow-up visit	No follow-up image
16	Acetaminophen	Improved within a few days	Resolved
17	NSAIDs	No follow-up visit	No follow-up image
18	NSAIDs	Improved within a few days	Not resolved^#^
19	NSAIDs	Improved at 1-month follow-up	Decreased
20	Acetaminophen	Improved at 2-week follow-up	No follow-up image
21	NSAIDs	Improved at 1-month follow-up	Decreased
22	NSAIDs	Improved within a few days	Decreased
23	NSAIDs	No follow-up visit	No follow-up image
24	NSAIDs	No follow-up visit	No follow-up image

*NSAIDs*, non-steroidal anti-inflammatory drugs

^#^Calcification has not resolved, but morphological change was observed

## Discussion

Paravertebral crystal deposition disease has previously been reported in only a few case studies, with its exact prevalence remaining unknown [9, 10]. Our investigation demonstrated that paravertebral crystal deposition, identified with a prevalence of 0.16% on CT scans of patients with torso pain, is a relatively rare condition but one that may be encountered in clinical practice. Radiologists should consider the possibility of paravertebral crystal deposition disease and diligently investigate the structures surrounding the vertebral bodies when interpreting CT scans that were performed to investigate the causes of trunk pain.

Crystal deposition diseases affecting the spine include pediatric intervertebral disc calcification, yellow ligament calcification, and calcific longus colli tendinitis. Intervertebral disc calcification is a characteristic condition that occurs in children, differing significantly from the demographics of the cohort presented in this study (6, 7). In contrast, calcific longus colli tendinitis typically presents with neck pain and involves calcification in the longus colli muscle at the C1-3 level [16]. Annulus fibrosus calcification, characterized by calcium deposits within the outer layers of the intervertebral disc, is commonly observed in elderly individuals, with increasing prevalence correlating with age [17]; this condition is typically asymptomatic.

Our study found that paravertebral crystal deposition disease predominantly affected the lower thoracic and lumbar spine, with calcification consistently located adjacent to the intervertebral disc in all cases along the axial direction. Notably, previous studies have reported that annulus fibrosus calcification occurs more frequently in the lower thoracic spine [17], which aligns with the high prevalence of paravertebral crystal deposition at the same levels in this study. Alternatively, given that calcification in crystal deposition diseases tend to migrate to surrounding tissues, the observed calcification may represent deposits that have moved from the annular fibrocartilage to the anterior longitudinal ligament or adjacent adipose tissue [18]. Considering the above, it is possible that paravertebral crystal deposition disease represents a subset of symptomatic cases of annulus fibrosus calcification.

Crystal deposition diseases, particularly calcium pyrophosphate deposition disease, often induce pain during the resorption phase [1, 19]. In our study, the maximum CT values of crystal deposition averaged approximately 800 HU, which is lower than those of cortical bone calcifications. Typically, calcifications are assessed using the bone window setting on CT. Therefore, when assessing calcifications, employing appropriate CT display settings is essential to avoid overlooking crystal deposits.

In this study, we included cases with soft tissue density around the vertebral body as part of the inclusion criteria. Two cases without calcification were excluded from the current cohort but did not meet the diagnostic criteria for other diseases, such as vertebral osteomyelitis. However, it remains possible that these cases represented paravertebral crystal deposition disease, with the CT performed either before the appearance of calcification or after its resolution.

The precise nature of the crystals in paravertebral crystal deposition disease has not been pathologically confirmed. Calcifications occurring in intervertebral discs are known to be either calcium pyrophosphate crystals or hydroxyapatite crystals, with calcium pyrophosphate being more commonly observed in the annulus fibrosus [20]. Paravertebral crystal deposition disease is generally managed with conservative treatment and follow-up, thereby making opportunities to obtain calcification samples rare. Non-invasive methods using dual-energy CT may assist in inferring the composition of these unknown crystal deposits in future research.

This study adds to the current knowledge base by ascertaining a prevalence rate of 0.16% for paravertebral crystal deposition disease among patients with trunk pain—which is often overlooked in clinical practice; furthermore, most affected patients were middle-aged women. It delineates CT imaging characteristics of the disease, such as calcifications primarily located in the lower thoracic and upper lumbar spine. These findings emphasize the need for clinicians to consider paravertebral crystal deposition disease in differential diagnoses when patients present with unexplained trunk pain. As a self-limiting disease, paravertebral crystal deposition responds well to symptomatic treatment, leading to the resolution of symptoms and the disappearance of calcifications on CT imaging. Understanding this condition could lead to improvements diagnostic accuracy and patient management, and future research should investigate the mechanisms of crystal deposition and refine diagnostic criteria to enhance outcomes.

While this study offers important insights, several limitations should be acknowledged. First, the single-center, retrospective nature of the study may have introduced inherent biases related to sample selection and patient demographics. Since all patients were of Asian descent, no conclusions can be drawn regarding differences in disease prevalence among different ethnic groups. Second, although we broadly analyzed patients with trunk pain as their chief complaint for CT evaluation, selection bias remains a possibility. This study relied on patients who were already referred for CT imaging, inherently excluding individuals with trunk pain who were either not referred for imaging or managed with alternative diagnostic methods. Broadening inclusion criteria to encompass a wider range of symptoms and diagnostic methods may help capture patients undergoing alternative diagnostic examinations besides CT imaging. Third, despite the substantial number of CT cases examined, the limited number of positive cases (*n* = 24) restricts the scope of analysis. Nevertheless, accurate diagnosis of this condition, detectable using CT, is crucial for radiologists to enable delivery of effective patient care. Fourth, among cases diagnosed with paravertebral crystal deposition disease, only a limited number had pre-onset and follow-up CT scans available, leaving gaps in our understanding of the origin and the natural history of this condition on imaging. Fifth, the origin of calcification and the composition of the crystals remain speculative as no direct evaluations have been performed. Future research should aim to address these limitations and further elucidate the clinical implications and pathophysiology of paravertebral crystal deposition disease.

In conclusion, paravertebral crystal deposition disease was identified in 0.16% of patients undergoing CT scans for torso pain. Characteristic CT findings included calcifications located primarily in the lower thoracic and upper lumbar spine, particularly in the anterior region of the vertebral body, accompanied by soft tissue swelling. Our findings highlight the importance of considering paravertebral crystal deposition disease in differential diagnoses for patients with unexplained torso pain—a common clinical scenario that could otherwise lead to misdiagnoses if not carefully assessed (Supplemental Fig. 2).

## Supplementary information

Below is the link to the electronic supplementary material.

ESM 1 (PNG 123 KB)

High Resolution Image (TIF 1.06 MB)

ESM 2 (PNG 400 KB)

High Resolution Image (TIF 2.17 MB)

## Data Availability

The datasets generated and analyzed during the current study are not publicly available due to privacy and ethical restrictions but are available from the corresponding author on reasonable request and with permission of the institutional review board.
