# Supplementary figures and images for: Paravertebral crystal deposition disease: a retrospective study of clinical presentation, prevalence, and CT imaging findings
Source: Skeletal Radiol. 2025 Jan 16;54(8):1643–51. doi: 10.1007/s00256-025-04874-w (PMC12174259; doi:10.1007/s00256-025-04874-w)

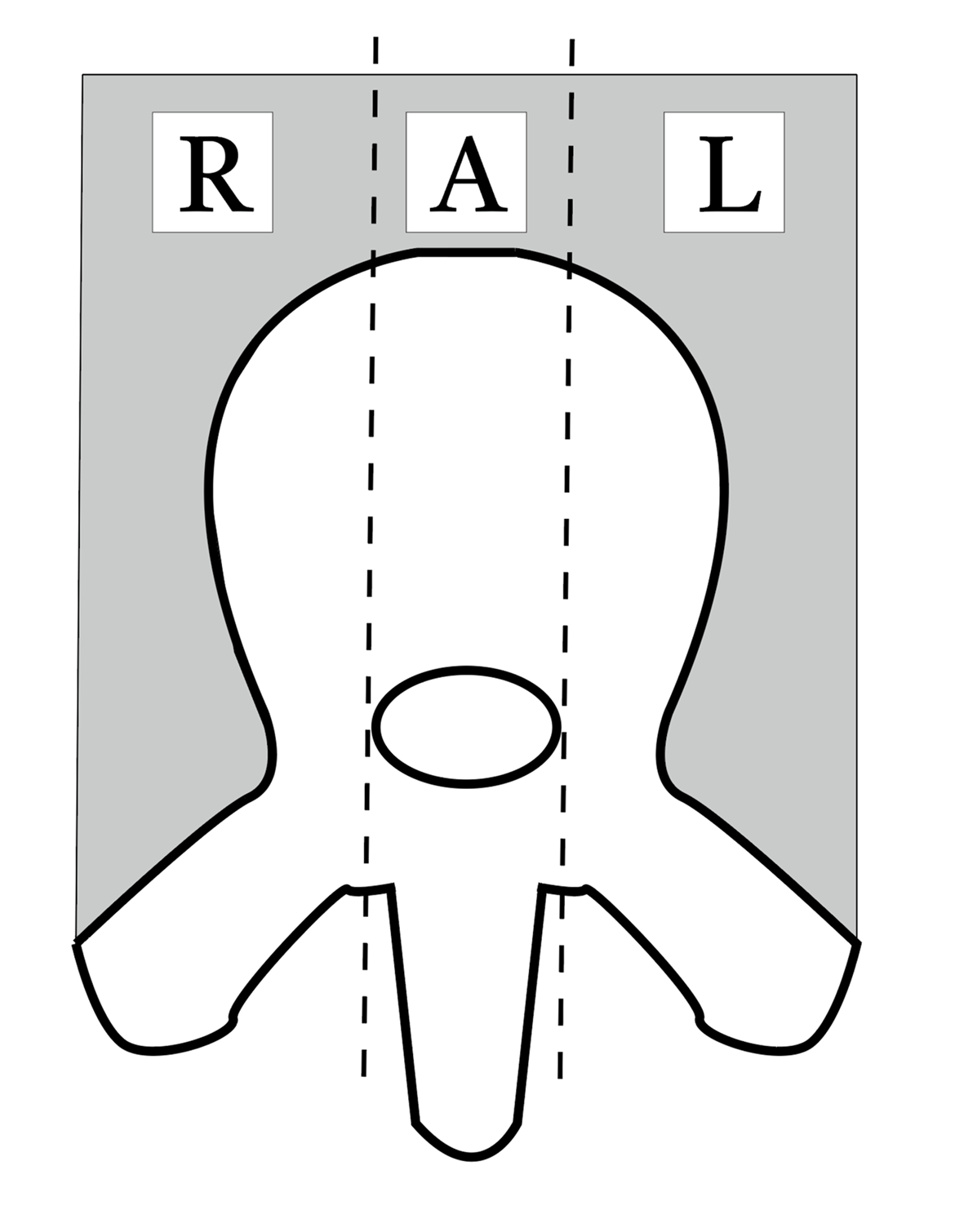

Supplement: Supplementary file 1 — (PNG 123 KB) [file 256_2025_4874_Fig5_ESM.png]

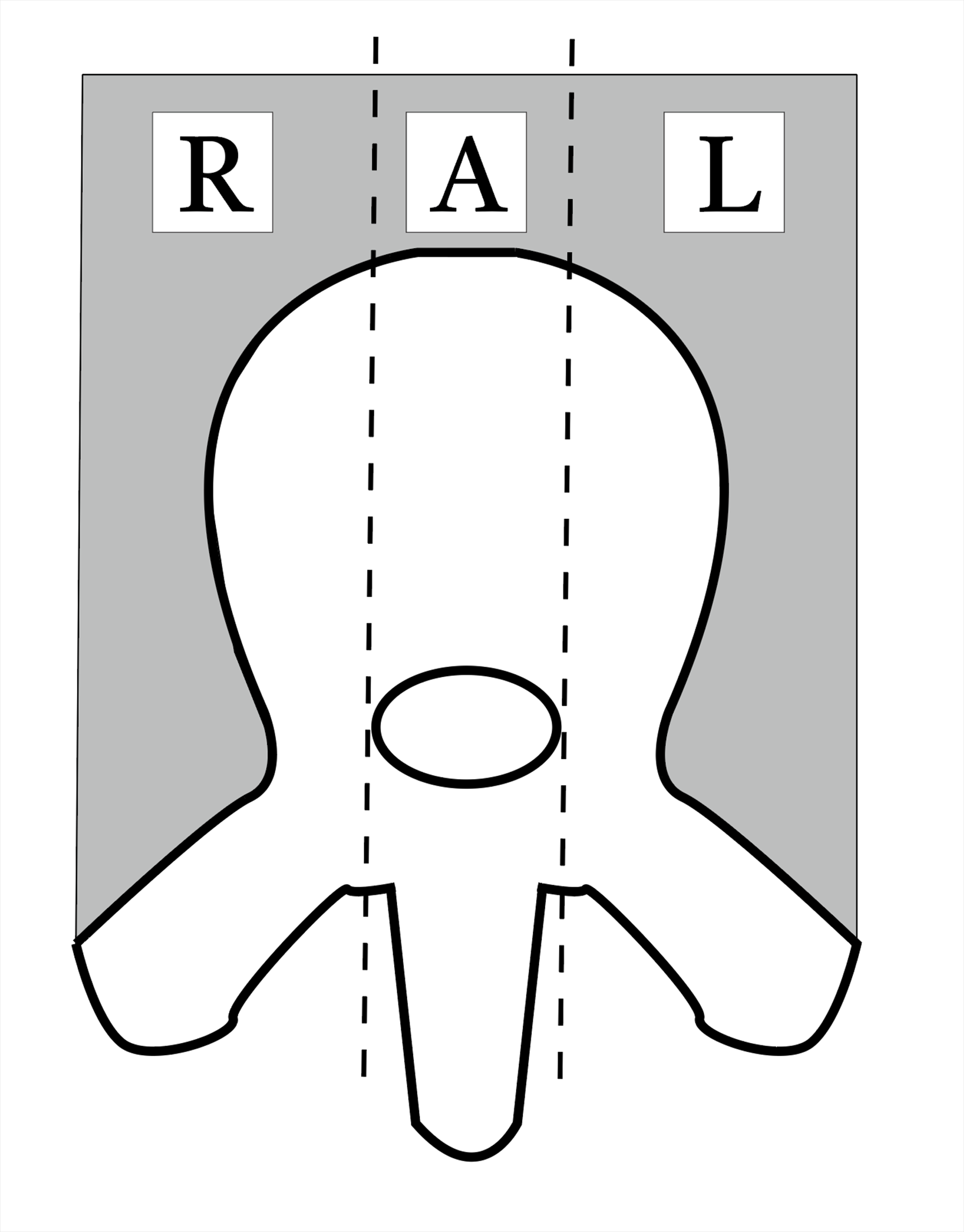

Supplement: Supplementary file 2 — High Resolution Image (TIF 1.06 MB) [file 256_2025_4874_MOESM1_ESM.tif]

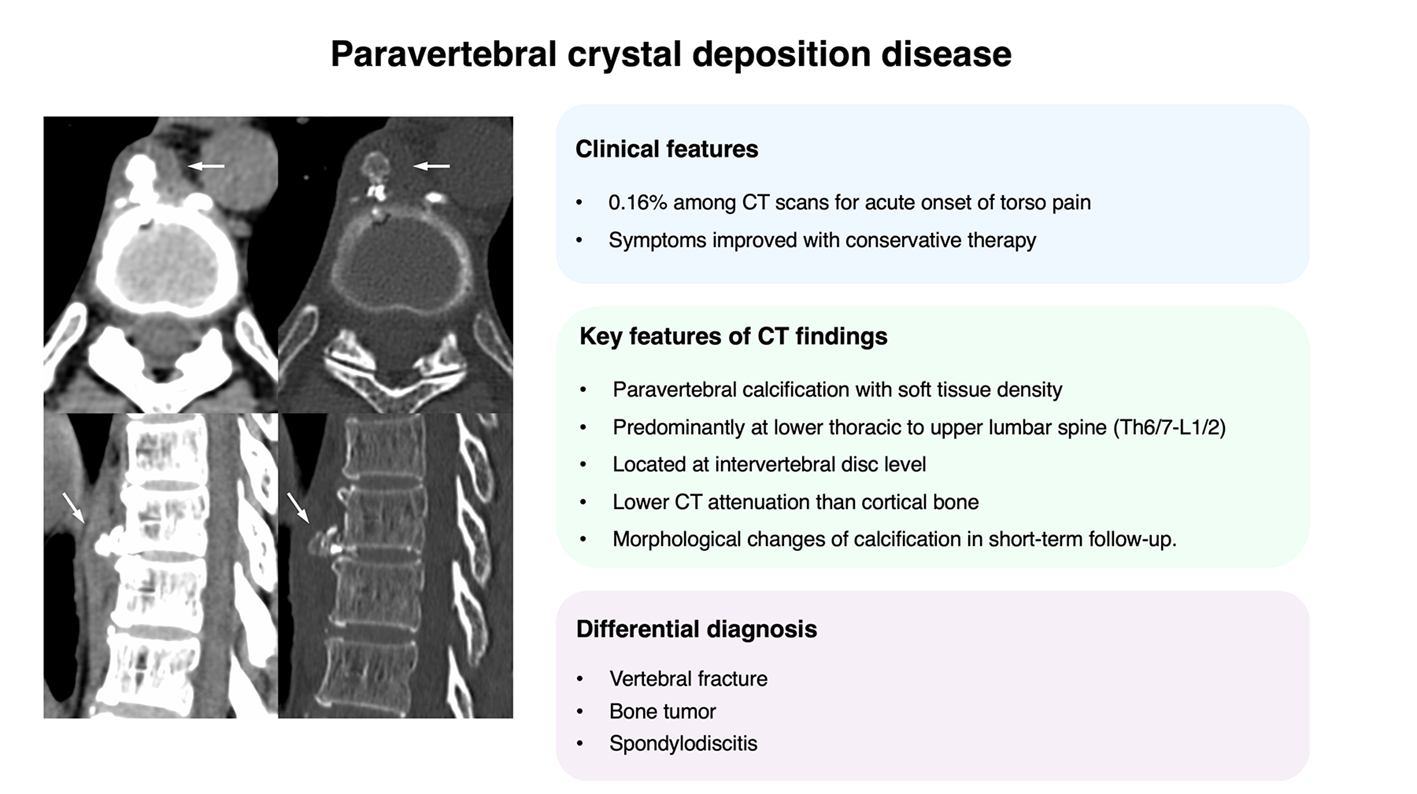

Supplement: Supplementary file 3 — (PNG 400 KB) [file 256_2025_4874_Fig6_ESM.png]

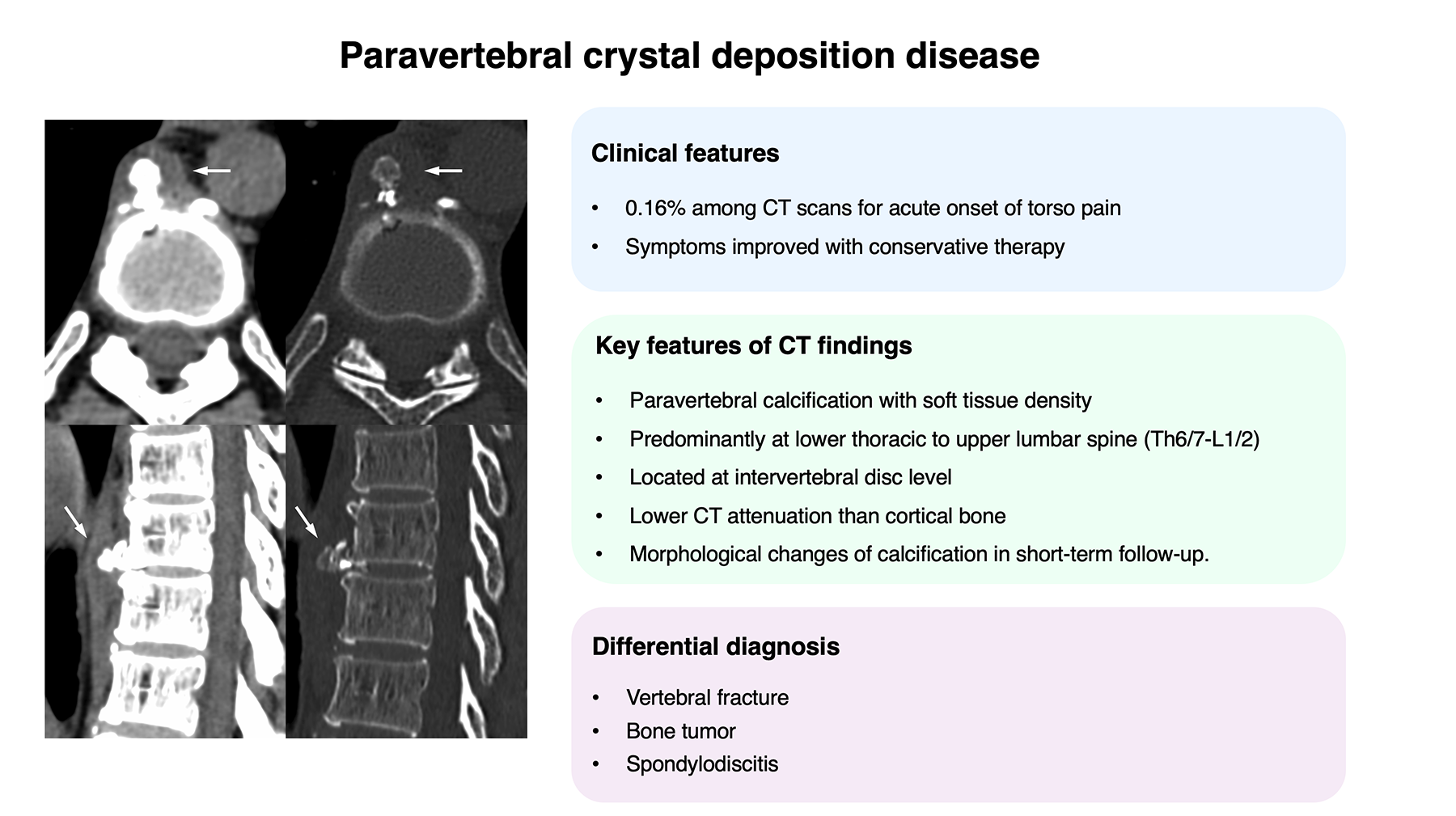

Supplement: Supplementary file 4 — High Resolution Image (TIF 2.17 MB) [file 256_2025_4874_MOESM2_ESM.tif]
